# Relative contribution of various chronic diseases and multi-morbidity to potential disability among Dutch elderly

**DOI:** 10.1186/s12913-017-2820-0

**Published:** 2018-01-15

**Authors:** Riaan Botes, Karin M. Vermeulen, Janine Correia, Erik Buskens, Fanny Janssen

**Affiliations:** 1 Clinical Epidemiology, University Medical Centre Groningen, University of Groningen, Groningen, the Netherlands; 2 Department of Epidemiology, University of Groningen, University Medical Center Groningen, Groningen, the Netherlands; 3 Department of Medical Biosciences, University of the Western Cape, Bellville, South Africa; 4 Population Research Centre, University of Groningen, Groningen, the Netherlands; 5 The Netherlands Interdisciplinary Demographic Institute, The Hague, the Netherlands

**Keywords:** Chronic disease, Elderly, Multi-state life tables, Sex and age specific interventions

## Abstract

**Background:**

The amount of time spent living with disease greatly influences elderly people’s wellbeing, disability and healthcare costs, but differs by disease, age and sex.

**Methods:**

We assessed how various single and combined diseases differentially affect life years spent living with disease in Dutch elderly men and women (65+) over their remaining life course. Multistate life table calculations were applied to age and sex-specific disease prevalence, incidence and death rates for the Netherlands in 2007. We distinguished congestive heart failure, coronary heart disease (CHD), breast and prostate cancer, colon cancer, lung cancer, diabetes, COPD, stroke, dementia and osteoarthritis.

**Results:**

Across ages 65, 70, 75, 80 and 85, CHD caused the most time spent living with disease for Dutch men (from 7.6 years at age 65 to 3.7 years at age 85) and osteoarthritis for Dutch women (from 11.7 years at age 65 to 4.8 years at age 85). Of the various co-occurrences of disease, the combination of diabetes and osteoarthritis led to the most time spent living with disease, for both men (from 11.2 years at age 65 to 4.9 -years at age 85) and women (from 14.2 years at age 65 to 6.0 years at age 85).

**Conclusions:**

Specific single and multi-morbid diseases affect men and women differently at different phases in the life course in terms of the time spent living with disease, and consequently, their potential disability. Timely sex and age-specific interventions targeting prevention of the single and combined diseases identified could reduce healthcare costs and increase wellbeing in elderly people.

## Background

The ultimate aim of healthcare should be to reduce disability and increase wellbeing [1]. Both disability and wellbeing are broad concepts, however. Because disability is usually defined as a limitation in physical or mental functioning, caused by the presence of disease [2], the amount of time spent with disease is an important factor to consider when determining both disability and wellbeing. The more time spent living with disease, the higher the level of potential disability experienced, and the higher the individual and healthcare related costs.

Reducing disability and the associated healthcare-related costs becomes even more challenging with the rapid ageing of populations in Western societies, particularly in Europe. Demographic projections indicate that 30% of the European population will be aged 65 or over by 2050 [3]. It is however clear that not all public health interventions aimed at addressing morbidity and longevity are effective [4]. Understanding which group of elderly people should be targeted by disability/disease prevention programmes is important for reducing the burden of highly-prevalent diseases and combating multi-morbidity among the elderly [5]. Timely interventions targeted at vulnerable groups may be able to alter undesirable health pathways and postpone disease development [6]. This warrants a closer look into differences by disease, age and sex in time spent living with disease and time spent living with comorbidity.

Many people aged 65 and older suffer from chronic multi-morbid conditions associated with increased disability and reduced health-related quality of life (HRQOL) [7]. Suffering from multi-morbidity also causes elderly people to use healthcare resources more often and to require more frequent hospitalization than when suffering from a single disease [8].

Multi-morbidity and type of single chronic disease influence to a large extent the time spent living with disease [9, 10]. Diseases like COPD, cardiovascular disease (CVD), dementia, diabetes and osteoarthritis are all diseases which have a considerable impact on elderly people’s disability and associated quality of life and frequently occur together [11]. Future projections indicate that high-income countries can expect ischemic heart disease to account for 5.9% of the total disability-adjusted life years by 2030. Other cardiovascular diseases and COPD will account for 4.5% and 2.5% of the total disability-adjusted life years in 2030 respectively [12].

Alongside chronic disease profiles including multi-morbidity, age and gender also play an important role in determining health transitions and the time spent living with disability in elderly people [13–16]. Health transition typically refers to transition from a healthy state to a diseased or disabled health state. It has been noted that fewer elderly women are in good health than men, yet women live longer than men [13]. Women are also more likely to suffer from multi-morbidity compared to men [13]. The experience of disease and disability from the perspective of ‘young’ elderly people might also be very different from older and very old elderly people. Therefore, age and sex adjusted outcomes are needed to effectively plan for healthcare services for the aging population [17].

Understanding the effect of different chronic diseases and their co-occurrence on morbidity across the elderly life course is essential to improve the provision of cost-effective treatment options and taking into consideration the variable effect of chronic disease on health transitions in the male and female populations at different older ages [17–19].

This study aims to assess how various single and multi-morbid conditions will influence life years spent living with disease for elderly in the Netherlands, thereby emphasizing differences between men and women and differences by age over the remaining life course.

## Methods

### Setting and data sources

We assessed the average remaining number of life years that are expected to be spent living with various single and combinations of diseases for Dutch men and women aged 65, 70, 75, 80 and 85 in 2007.

Table 1 lists the specific diseases and disease combinations we included in our study. The specific diseases were chosen because they were the most prevalent within the Dutch elderly population [20–22]. The disease combinations were included to demonstrate the effects of the combination of potentially fatal diseases (CVD, cancer and COPD) and the combination of mostly non-fatal diseases (osteoarthritis, dementia and diabetes) (29;30). In doing so, a maximum of three diseases were combined.

**Table 1:** Single and Multi-morbid disease combinations

Single disease	Multi-morbid disease
Congestive heart failure (CHF)	CHF + CHD
Coronary heart disease (CHD)	Dementia + stroke
Breast cancer (women only)	Diabetes + osteoarthritis
Prostate cancer (men only)	CHF + osteoarthritis
Colon cancer	CHF + CHD + diabetes
Lung cancer	Dementia + stroke + CHF
Diabetes	Dementia + stroke + lung cancer
Chronic obstructive pulmonary disease (COPD)	Dementia + stroke + colon Cancer
Stroke	Dementia + stroke + prostate cancer
Dementia	Diabetes + osteoarthritis + dementia
Osteoarthritis	CHF + osteoarthritis + COPD

We obtained the health data below on the total population in the Netherlands in 2007 by age (0–4, 5–9, …, 80–84, 85+) and sex. Population numbers and all-cause and cause-specific death numbers were obtained from Statistics Netherlands. Disease incidence rates and disease prevalence were obtained from the National Institute of Public Health and the Environment [23]. The data were freely available to the public and, according to Dutch legislation, no ethical approval was necessary to perform the research.

### Multistate life tables calculations

We applied multistate life table calculations to each disease and each disease combination. Multistate life table calculations (often referred to as multistate life tables) are an important demographic tool used to estimate the expected average time spent in a given state from a particular age, in our example the time spent living with and without a particular disease (or disease combination). Essentially, a multistate life table is an extension of the general life table in which the expected (remaining) number of years of life (life expectancy) is assessed based on age-specific mortality rates [24]. Multistate life tables, however, compare more states than life and death, and more transitions than just dying/mortality, and use age-specific transition rates linked to the various transitions as input for the calculations. In our case, we considered three states: 1) without a particular disease or disease combination (non-diseased), 2) with a particular disease or disease combination (diseased), and 3) death. We then distinguished three transitions: 1) mortality from non-diseased to death, 2) mortality from diseased to death, and 3) diseased from non-diseased. We used the relevant age and sex-specific transition rates as input: 1) mortality rates in the non-diseased population calculated by dividing the all-cause death numbers by the non-diseased population, 2) mortality rates for the diseased population calculated by dividing the cause-specific death numbers by the diseased population, and 3) the disease-specific incidence rates. The diseased population was calculated by multiplying disease prevalence by the total population, and the non-diseased population was obtained by subtracting the diseased population from the total population.

Since the diseases considered are generally chronic, we assumed no recovery and thus excluded the transition from diseased to non-diseased.

We followed the life table calculations as described in detail by Nusselder and Peeters [5, 25], which include the following steps: 1) putting the rates in a matrix format for each age, 2) transforming the age-specific rate matrices to age-specific probability matrices, 3) using information from the age-specific probability matrices as input for the two life tables: one referring to the disease state and the other to the non-diseased state, and 4) applying the normal life table calculations to the two life tables to obtain the average remaining number of years spent living either with or without the disease.

Like previous studies, we assumed that transition rates were constant across the 5-year intervals. We applied the life table calculations to five-year age groups starting at age 0, and assumed that no one suffered from the studied diseases and disease combinations at birth. For the number of years spent living in the open ended age group for the various states we used life expectancy at age 85 in 2007 from Statistics Netherlands: 5.3 years for men and 6.6 years for women.

Multi-morbidity was estimated by combining single disease transition rates by simple addition, without interactions [21, 26]. For example, we estimated the multi-morbidity of CHD and CHF by adding (1) the CHD incidence rate to the CHF incidence rate, (2) the CHD diseased death rate to the CHF diseased death rate, and (3) the CHD non-diseased death rate to CHF non-diseased death rate. Using the combined transition rates as input for the multistate life table calculations, we obtained the average number of years Dutch men or women aged 65, 70, 75, 80, and 85 can expect to live with CHD and CHF combined.

## Results

In 2007 Dutch men and women aged 65 could expect to live another 17.4 and 20.9 years on average, respectively. Of these remaining years, more years – compared to other diseases – will be spent living with either osteoarthritis (7.1 years for men, 11.7 years for women), diabetes (7.0 and 6.5 years, respectively) and CHD (7.6 and 5.0 years, respectively (Table 2). The same applies to Dutch people at older ages in 2007, although clear sex differences appear. Men can expect to spend the most years with CHD, starting from 7.6 years at age 65 to 3.7 years at age 85. Women can expect to spend the most remaining years with osteoarthritis, starting from 11.7 years at age 65 to 4.8 years at age 85.

**Table 2:** Total remaining life expectancy and remaining life expectancy spent living with different single diseases (in years), for Dutch men and women aged 65, 70, 75, 80 and 85 in 2007

Remaining life expectancy spend with a certain disease^a^											
Age	Remaining life expectancy^b^	Diabetes	CHF	CHD	COPD	Osteoarthritis	Dementia	Lung Cancer	Colon Cancer	Prostate/Breast Cancer	Stroke
Men											
65	17.4	7.0	2.3	7.6	3.7	7.1	0.6	0.1	0.6	1.6	2.3
70	13.6	6.4	2.3	7.0	3.4	6.6	0.6	0.1	0.6	1.5	2.2
75	10.3	5.7	2.3	6.2	3.0	6.0	0.6	0.1	0.5	1.4	2.0
80	7.5	4.7	2.1	5.2	2.5	5.0	0.6	0.1	0.4	1.2	1.8
85	5.3	3.2	1.7	3.7	1.9	3.3	0.5	0.0	0.3	0.9	1.4
Women											
65	20.9	6.5	2.1	5.0	4.2	11.7	1.0	0.1	0.5	1.6	2.1
70	16.8	5.9	2.0	4.7	3.7	10.6	1.0	0.1	0.4	1.4	1.9
75	12.9	5.1	1.9	4.2	3.1	9.2	1.0	0.0	0.4	1.2	1.7
80	9.5	4.2	1.8	3.5	2.5	7.3	0.9	0.0	0.3	0.9	1.5
85	6.6	3.0	1.4	2.6	1.8	4.8	0.7	0.0	0.2	0.7	1.1

^a^The average number of remaining life years Dutch men or women in 2007 at the specified age can expect to live with a certain disease

^b^The average number of remaining life years Dutch men or women in 2007 at the specified age can expect to live

The share of remaining life time spent with disease increases significantly from one age group to the next for certain diseases, see Table 3.

**Table 3:** Share of remaining life time spent living with different single diseases (in percentages), for Dutch men and women aged 65, 70, 75, 80 and 85 in 2007

Age	Diabetes	CHF	CHD	COPD	Osteoarthritis	Dementia	Lung Cancer	Colon Cancer	Prostate Cancer	Stroke
Men										
65	40.2	13.3	43.4	17.8	40.7	3.5	0.8	3.4	9.2	13.2
70	47.0	17.0	51.3	20.3	48.6	4.7	0.9	4.1	11.3	16.0
75	55.0	21.9	60.5	22.7	57.9	6.3	0.9	4.9	13.5	19.6
80	62.8	27.8	69.7	24.9	66.8	8.5	0.8	5.7	15.9	24.2
85+	60.4	31.5	69.2	26.2	63.0	10.2	0.7	6.2	17.3	27.1
Women										
65	31.0	9.9	24.0	20.1	55.8	4.7	0.3	2.3	7.8	9.8
70	34.9	12.0	27.9	22.1	63.1	5.9	0.3	2.6	8.4	11.5
75	39.5	15.0	32.5	24.2	71.1	7.6	0.3	3.0	9.0	13.5
80	43.8	18.6	37.1	26.2	76.9	9.5	0.2	3.4	9.7	15.6
85+	45.2	21.4	39.8	27.4	73.1	11.1	0.2	3.6	10.3	17.3

The share of remaining life time spent with diabetes, CHD and osteoarthritis increases significantly for men from one age group to the next, whereas this is only true for women with osteoarthritis. The share of remaining life time spent living with stroke, dementia, colon cancer, prostate/breast cancer and lung cancer does not change much over the life course for men and women.

When considering the combination of diseases, older Dutch men and women in 2007 could expect to live most of their remaining life years with the ‘Diabetes + osteoarthritis’ disease pair (Table 4). Dutch men aged 65 in 2017 can expect to live 11.2 years with diabetes and osteoarthritis combined, and Dutch women aged 65, 14.2 years. At age 85, the figures are 4.9 years for men and 6.0 years for women, respectively.

**Table 4:** Total remaining life expectancy and remaining life expectancy spent living with different combinations of diseases (in years), for Dutch men and women aged 65, 70, 75, 80 and 85 in 2007

Remaining life expectancy spend with a certain disease^a^												
Age	Remaining life expectancy^b^	CHF + CHD	Dementia + stroke	Diabetes + Osteoarthritis	CHF+ Osteoarthritis	CHF + CHD+ Diabetes	Dementia + stroke + CHF	Dementia + Stroke +lung cancer	Dementia +stroke +colon cancer	Dementia +stroke +Prostate /breast Cancer	Diabetes + osteoarthritis + dementia	CHF + osteoarthritis + COPD
Men												
65	17.4	5.8	1.8	11.2	5.5	7.0	1.9	0.3	1.0	1.8	5.2	4.8
70	13.6	5.6	1.8	10.6	5.4	6.9	2.0	0.3	1.0	1.8	4.9	4.6
75	10.3	5.4	1.8	9.6	5.3	6.6	2.1	0.2	1.1	1.8	4.6	4.4
80	7.5	5.2	1.9	7.9	5.0	6.2	2.5	0.2	1.3	2.1	4.7	4.2
85	5.3	5.0	2.1	4.9	4.3	5.2	3.8	0.4	2.2	3.2	5.0	4.9
Women												
65	20.9	4.8	2.3	14.2	9.1	6.8	2.4	0.4	1.3	2.2	7.9	8.6
70	16.8	4.7	2.2	12.9	8.4	6.4	2.4	0.4	1.3	2.1	7.1	7.9
75	12.9	4.5	2.1	11.2	7.6	6.0	2.4	0.4	1.3	2.0	6.2	7.2
80	9.5	4.2	2.0	9.0	6.5	5.6	2.5	0.4	1.3	1.9	5.6	6.4
85	6.6	3.9	1.9	6.0	5.3	5.4	3.1	0.5	1.6	2.4	5.7	5.9

^a^The average number of remaining life years Dutch men or women in 2007 at the specified age can expect to live with a certain disease

^b^The average number of remaining life years Dutch men or women in 2007 at the specified age can expect to live

Again, important sex differences appear. Men can expect to spend more years with the ‘CHF + CHD’, ‘diabetes + osteoarthritis’ and ‘CHF + CHD + diabetes’ disease combinations, while women can expect to spend more years with ‘CHF + osteoarthritis’, ‘diabetes + osteoarthritis’ and ‘CHF + osteoarthritis + COPD’.

The proportion of remaining life years spent with disease also increases progressively from one age group to the next for the different combinations of diseases (Table 5). The exceptions are dementia and stroke and cancer combinations, where both men and women will spend a similar percentage of their remaining life years with disease from one age group to the next.

**Table 5:** Share of remaining life time spent living with different combinations of diseases (in percentages), for Dutch men and women aged 65, 70, 75, 80 and 85 in 2007

Age	CHF + CHD	Dementia + stroke	Diabetes + Osteoarthritis	CHF+ Osteoarthritis	CHF + CHD+ Diabetes	Dementia + stroke + CHF	Dementia + Stroke +lung cancer	Dementia +stroke +colon cancer	Dementia +stroke +Prostate /breast Cancer	Diabetes + osteoarthritis + dementia	CHF + osteoarthritis + COPD
Men											
65	33.3	10.3	64.4	31.6	40.2	10.9	1.7	5.7	10.3	29.9	27.6
70	41.2	13.2	77.9	39.7	50.7	14.7	2.2	7.4	13.2	36.0	33.8
75	52.4	17.5	93.2	51.5	64.1	20.4	1.9	10.7	17.5	44.7	42.7
80	69.3	25.3	105.3	66.7	82.7	33.3	2.7	17.3	28.0	62.7	56.0
85	94.3	39.6	92.5	81.1	98.1	71.7	7.5	41.5	60.4	94.3	92.5
Women											
65	23.0	11.0	67.9	43.5	32.5	11.5	1.9	6.2	10.5	37.8	41.1
70	28.0	13.1	76.8	50.0	38.1	14.3	2.4	7.7	12.5	42.3	47.0
75	34.9	16.3	86.8	58.9	46.5	18.6	3.1	10.1	15.5	48.1	55.8
80	44.2	21.1	94.7	68.4	58.9	26.3	4.2	13.7	20.0	58.9	67.4
85	59.1	28.8	90.9	80.3	81.8	47.0	7.6	24.2	36.4	86.4	89.4

Despite the generally much lower share of remaining life years spent living with disease at age 85 compared to age 80, the share of remaining life years spent living with disease combinations including dementia and stroke actually increases from age 80 to age 85.

## Discussion

Across ages 65 and over, CHD caused the most time spent living with disease for Dutch men and osteoarthritis for Dutch women. Of the various co-occurrences of disease, the combination of diabetes and osteoarthritis led to the most time spent living with disease, for both Dutch men and women aged 65 and over.

Disease type and disease prevalence appear to be important factors when determining time spent living with disease by elderly Dutch men and women.

Intuitively, diseases classified as non-fatal will cause elderly men and women to spend more time living with disease and disability and thus require more healthcare resources, especially when these diseases are highly prevalent. Osteoarthritis is not only considered non-fatal but is also highly prevalent among Dutch elderly. Especially for elderly female patients osteoarthritis proved important in terms of the time spent with disease. Eliminating osteoarthritis and other non-fatal disorders would result in savings in hospital care and nursing and residential care facilities [27].

The fatal disease CHD causing the most time spent living with disease for Dutch men could also be due to the high prevalence of CHD in elderly men, but is also the result of effective treatment, i.e. the increased healthcare resources allocated to the management of cardiovascular disease in the preceding years. This explanation is in line with the considerable improvement of the survival rate of elderly CHD patients [28]. Also, diabetes is a highly prevalent disease among elderly Dutch people, but not necessarily fatal if controlled properly, which could account for the increased time spent living with disease by elderly diabetes patients. Clearly, the elimination of highly fatal diseases such as CHD but also neoplasms will not only result in a decrease in hospital care costs, but also in an increase in time spent living with the disease, and consequently increasing nursing and residential care facilities costs.

Our results not only clearly indicate important differences between men and women in the impact of specific individual and combined diseases, but also clear differences in their impact by age across the remaining life course at age 65. The time Dutch elderly men spend with either diabetes, CHD or osteoarthritis increases progressively with age, and similarly for Dutch elderly women with osteoarthritis. The various cancers, stroke and dementia, however, do not show the same increasing trend of disability over the life course of elderly women. This clearly indicates that specific single diseases affect elderly men and women differently at different phases in the life course in terms of the time spent living with disease, and consequently, their potential disability and quality of life. These important differences need to be considered when planning for healthcare and when designing interventions.

As far as multi-morbidity is concerned our results indicate that both diabetes and osteoarthritis can be considered non-fatal diseases and the combination of these two diseases can increase the time spent with disease. Clearly, as far as the effect of multi-morbidity on morbidity is concerned, the non-fatal combination of diabetes and osteoarthritis significantly reduces the disability-free period elderly men and women will enjoy.

The multi-morbid disease combinations which include dementia and stroke appear to be particularly ‘oldest old’ problems, since they increase the time men and women spend with disease in the 80–84 and 85+ age groups.

Oostrom et al. have shown that individuals suffering from multi-morbidity receive more face-to-face and telephone consultations with general practitioners, more minor operations, increased use of prescription medication, more home visits and more referrals to specialized care [29]. People with multi-morbidity may be receiving improved treatment of their known conditions, which might also result in early detection of additional diseases, increasing the survival rate of elderly with multi-morbidity and extending the time spent with disease.

We infer that, aside from the effects on health resource utilization, elderly people with multi-morbidity may also be receiving better management of their multi-morbid conditions because they use healthcare services more frequently, effectively altering their disease progression and postponing mortality [30].

Since the prevalence rates of multi-morbid disease are not readily available it is uncertain whether and to what extent the prevalence rates of multi-morbid diseases contribute to the time elderly Dutch people spend with disease. However, it follows our choice to combine transition rates to estimate multi-morbid conditions that we should assume that the prevalence of diseases and the combinations thereof proportionally affect the time spent with disease.

Understanding the effects of multi-morbid diseases on the elderly male and female population could help decision-makers plan appropriate pro-active and timely interventions early in life to negate the negative effects of multi-morbidity in later life.

In sum, information from studies like ours provide an indication for sex- and age-specific interventions aimed at the identified individual and combined diseases that cause the most time spent living with disease by age and sex, with as the ultimate aim to decrease disability across the remaining life course of the elderly. Identifying and acknowledging the effects of specific disease on elderly disability is only the first step in a remedial process. Conceivably diseases like diabetes, osteoarthritis and CHD can be cost-effectively managed or even avoided by altering unhealthy lifestyle choices, i.e. doing exercise or by making healthy dietary choices. Identifying and understanding the social, cultural and economic barriers that prohibit individuals from making or adhering to healthy lifestyle choices, is however essential as well to address the disability associated with specific diseases [31].

Educating health care services regarding important elderly disease interactions and their implications can enhance the effectiveness of interventions to diminish disability [32].

### Strengths and limitations

A multi-state life table approach was used in this study to provide an overview of the disability caused by disease and multi-morbidity. A strength of this approach is the use of data to provide a prospective view on disease progression and disability projections. Although the use of combined transition rates to simulate multi-morbid conditions is a simplification of the multi-morbidity interactions, and 2007 data was implemented in this study, the results of this study are supported by subsequent studies, making the results significant and contributing to the expansion of knowledge in this research domain [33].

Firstly, the impact of the results can be substantial if utilized by clinicians and other stakeholders, along with results from similar studies within the healthcare sector to plan cost-effective interventions for the current elderly population. Preventive strategies for specific diseases, as indicated by our results, can provide better disability outcomes for the elderly and even delay the onset of disability. Secondly, studies like ours can assist decision-makers with difficult health resource allocation decisions for future elderly populations.

Understanding how elderly men and women within defined age groups will be affected by disease and multi-morbidity is a valuable tool to provide effective and relevant healthcare services to the elderly.

## Conclusions

Specific single and multi-morbid diseases affect elderly men and women differently at different phases in the life course in terms of the time spent living with disease, and consequently, their potential disability. Disease prevalence, disease type and disease interactions are important factors in this regard.

Cost effective interventions and specialized treatment regimens aimed at addressing specific diseases with a high prevalence and multi-morbidity could increase elderly people’s quality of life, while reducing disability and healthcare costs for the elderly population.

## Abbreviations

CHD: Coronary heart disease
CHF: Congestive heart failure
COPD: Chronic obstructive pulmonary disease
CVD: Cardiovascular disease
HRQOL: Health related quality of life
QoL: Quality of life
